# Demography and determinants of dog and cat ownership in three towns of West Shoa zone, Oromia Region, Ethiopia

**DOI:** 10.1186/s12917-020-02699-4

**Published:** 2020-12-10

**Authors:** Endrias Zewdu Gebremedhin, Edilu Jorga Sarba, Abraham Mekebib Getaneh, Getachew Kebebew Tola, Solomon Shiferaw Endale, Lencho Megersa Marami

**Affiliations:** 1 Department of Veterinary Science, College of Agriculture and Veterinary Sciences, Ambo University, P.O.Box 19, Ambo, Ethiopia; 2 Department of Veterinary Laboratory Technology, College of Agriculture and Veterinary Sciences, Ambo University, P.O.Box 19, Ambo, Ethiopia

**Keywords:** Dog, Cat, Demography, determinants, Ethiopia, Health risks, Ownership

## Abstract

**Background:**

The dog and cat population data is generally scarce in developing countries due to absence of surveys. The demography of owned dogs and cats, and the associated ownership characteristics, are essential for the control of pet population and zoonosis. This study was conducted in three towns of West Shoa Zone of Ethiopia with the objectives of assessing demographic characteristics of owned dogs and cats and determinants of ownership.

**Results:**

About 65.1% (95% CI: 62.1–69.8%) of the householders own dogs, 39.2% (95% CI: 35.8–43.8%) own cats, and 30.6% (95% CI: 27.4–35.0%) own both. The majority of the dog-owning households own a single dog (74.8%) and cat (74.9%). There were significantly higher proportion of dog and cat-owning households in Bako than Ambo and Gojo towns. The human to owned-dog ratio was 6:1, and that of cat ratio was 10:1. There were more male dogs (72.1%) and more female cats (59.7%). The male to female sex ratio was estimated at 3:1 for the dog while nearly 1:1 for cats. About 37.5% of the owned dogs were indoor, and 62.5% have free access to outside. Dogs and cats were acquired as a gift from families, neighbors, and friends. The identified reason for not owning dogs/cats were fear of zoonosis, dislike, no time to devote, benefit not realized, and shortage of finance. Logistic regression analysis identified study town, community type, gender of head of household, ownership of other animals as determinants for dog/cat ownership. Besides, possessing dogs was significantly associated with cat ownership.

**Conclusion:**

The current study provide insights into the determinants of dogs/cats ownership and their demographic characteristics in Ethiopia. Dogs are more commonly owned, but the household determinants for dog and cat ownership were comparable. Means of obtaining and reason of owning or abandoning dogs/cats is partly different from those reported in the developed countries. The results of this study could be used for the provision of veterinary services, quantifying health risks and benefits associated with dog/cat ownership, and control of pet population and related zoonosis.

**Supplementary Information:**

The online version contains supplementary material available at 10.1186/s12917-020-02699-4.

## Background

Dogs (*Canis familiaris*) and cats (*Felis catus*) are the most widespread and abundant carnivore animals in many parts of the world including Ethiopia. They are highly dependent on humans or human activities [1]. Domestic dogs and cats are often regarded as faithful friends and close companions of humans, and enjoy life together with humans [2]. This human-animal bond can provide significant positive benefits concerning emotional development and socialization. Studies have confirmed the mental and physical benefits of pet ownership and companionship, particularly among children, the elderly, and immune-compromised individuals [3].

In Ethiopia and elsewhere in the world free-roaming and uncontrolled dog and cat populations are reservoirs and transmitters of many zoonotic diseases including rabies [4–6], toxoplasmosis [7, 8] leishmaniasis [9], echinococcosis [10], toxocariasis [11], and ectoparasite infections [12, 13]. Moreover, road traffic accidents, bite injuries, noise during the night, predation, and competition with wildlife are the other challenges of dog and cat overpopulation [14]. The risk of environmental contamination with pathogens, exposure to accidents, welfare problems, and some infectious diseases will be more likely to unowned dogs and cats when compared to owned dog and cat populations [15]. Due to inadequate food provision, most owned dogs and cats are free-roaming in developing countries, which could serve as a source of infection for livestock and humans.

In Ethiopia, much of the emphasis is on food animals and well-documented data on dog and cat populations is unavailable [16]. However, in Addis Ababa dog population is estimated to be 250,000 to 350,000 of which half of the dog population may be owned [17]. No data is available on the size of the cat population.

Like many African countries, the rate of urbanization in Ethiopia is increasing rapidly and closely linked with human and dog populations. Therefore, understanding of dog and cat populations and associated ownership characteristics of these expanding urban communities remains a high priority [18]. Despite this presumably large number of dog and cat populations and the burden of zoonotic diseases in Ethiopia, research on determinants of their ownership as well as non-ownership is absent. Consequent to uncontrolled populations of dogs and cats living near the increasing densities of human populations, effective control of canine and feline originated zoonotic disease is an extremely challenging task. Knowledge of the determinants of ownership and demographic features of dogs and cats is essential in assessing the risk of disease transmission, promotion of responsible ownership, and planning of effective prevention and control of zoonotic diseases originating from dogs and cats. This study is a part of a larger study on determinants of dog and cat ownership and surveillance of diseases of public health importance in West Shoa Zone. The objectives of this study were to assess the determinants of dog and cat ownership and estimate the proportion of people owning dogs and cats.

## Results

### Socio-demographic characteristics of owned dogs and cats

Six hundred and ten households consisting of 305 in Ambo, 182 in Bako, and 123 Gojo were interviewed. Three hundred ninety-seven (65.1, 95% CI: 62.1–69.8%) of the householders owned dogs, whereas 239 (39.2, 95% CI: 35.8–43.8%) of them owned cats and 187 (30.6, 95% CI: 27.4–35.0%) owned both cats and dogs. Of the total 397 dog-owning households, 296 (74.8%) own a single dog, 76 (19.1%) owned two dogs, 20 (5.0%) owned three dogs and the remaining 5 (1.3%) owned four to six dogs. Likewise, of the 239 cat-owning households 179 (74.9%) owned one cat, 38 (15.9%) owned two cats, 18 (7.5%) owned three cats and the remaining 4 (1.7%) owned four to five cats. The average number of dogs owned by households (1.1) was not significantly different across the three towns (F = 0.976, *P* = 0.378). The same holds for the average number of cats owned by households. For Bako, Ambo, and Gojo towns the human to owned-dog ratio was 5:1, 6:1, and 9:1, respectively, the overall was 6:1. Similarly, that of cats was 11:1; 8:1, and 12:1, respectively, and the overall was 10:1. The proportion of dog-owning households was significantly higher in Bako town (75.8%) compared to Ambo (64.9%) and Gojo (49.6%) towns (Chi-square = 22.2, *P* ≤ 0.001). Likewise, the proportion of cat-owning households was significantly higher in Bako town (49.4%) compared to Ambo (36.7%) and Gojo (30.1%) towns (Chi-square = 13.1, *P* = 0.001). There were more male dogs (72.1%) and more female cats (59.7%). The male to female sex ratio for the dog was estimated to be 3:1 while it is nearly 1:1 for cats. According to the estimates of the interviewees, the maximum mean life expectancy of owned dogs was 12 years, and that of the cat was 9 years and there was no variation between the towns. There was no such variation in the mean estimated life expectancy/age of dogs and cats in the households of the three towns. The way of life of dogs from 37.5% (*n* = 149) of the dog-owning households was fully indoor and dogs are tied or confined in the garden, and 62.5% (*n* = 248) had either full or partial access to the outside/outdoor environment. Almost all of the owned cats had also outdoor access at least to the neighbor. The majority of the households own indigenous dogs (81.1%), while 17.6% own either exotic or cross, and a few (1.2%) own both. Data on the ownership characteristics of the owned dog and cat populations are shown in Table 1.

**Table 1:** Characteristics of dog populations in the three towns of West Shoa Zone, Ethiopia

Characteristics	Ambo		Bako		Gojo		Total	
	Dog	Cat	Dog	Cat	Dog	Cat	Dog	Cat
Number of respondents	305		182		123		610	
Ave. Family size	5.3		5.2		5.6		5.4	
Number of pet keeping HH	198	112	138	90	61	37	397	239
Proportion of pet owning HH	64.9	36.7	75.8	49.4	49.6	30.1	65.1	39.2
Human to pet ratio	8:1	12:1	6:1	10:1	11:1	17:1	7:1	12:1
Ave. number of pets per HH	0.7	0.4	0.9	0.5	0.5	0.3	0.7	0.4
Number of male pets	163	54	110	38	47	18	320	110
Number of female pets	52	80	52	60	20	23	124	163
Male to female sex ratio	3:1	1:1	2:1	1:1	2:1	1:1	3:1	1:1
Ave. No. of pets per owning HH	1.1	1.2	1.2	1.1	1.1	1.1	1.1	1.1
Ave. Number of pets per HH	1.3	1.4	1.4	1.3	1.3	1.4	1.4	1.4
Estimated pet’s life expectancy	12	9	12	10	12	9	12	9
Ave. Length of pet ownership	7.9		7.9		5.7		7.0	
No. of indoor dogs	98		34		17		149	
No. of partly/fully outdoor dogs	100		104		44		248	

*Ave.* Average, *HH* household, *No.* Number

### Acquisition, selection, the purpose of keeping and population control of dogs and cats

Sex of dog was the most important factor considered to select dogs (69.0%) followed by the color (49.1%), age (34.5%), breed (27.7%), and behavior (2.5%). Similarly, sex (39.7%), color (24.7%), age (20.9%), and breed (10.5%) were factors considered to select cats. Dogs were acquired as a gift from neighbors (46.3%), families (30.1%), and friends (13.4%), from the street (9.6%), and through purchase from a breeder (2.7%). Similarly, cats were acquired as a gift from neighbors (52.3%), families (24.3%), friends (9.2%), and as a stray from the street (7.3%), and breeder or purchased (0.8%). Dogs in the study towns were kept for multi-purpose and about 75.3% of the owned dogs were considered guard dogs for protection of household property, while 73.2% were also for love and affection and 33.0% were for companionship. Likewise, most owned cats were used for the protection of property from mice (83.7%), and companionship (43.9%) [Table 2].

**Table 2:** Means of acquiring, factors considered, and reason for keeping dogs and cats in the three study towns

Items	Category	Dog		Cat	
		No. of HH	Percent	No. of HH	Percent
Factors considered to select pet	Sex	274	69.0	95	39.7
	Color	195	49.1	59	24.7
	Age	137	34.5	50	20.9
	Breed	110	27.7	25	10.5
	Behavior/ aggressiveness	8	2.5	–	–
	Non responding	–	–	111	46.4
Means of acquiring the pet	Neighbours	184	46.3	105	43.9
	Family	111	27.9	72	30.1
	Friends	53	13.4	22	9.2
	Street breeder	38	9.6	18	7.5
	Breeder or purchase	11	2.7	2	0.8
The purpose of keeping the pet	Protection of property	304	76.5	200	83.7
	Love and affection	116	29.2	–	–
	Companionship	131	33.0	105	43.9

Pet/s = dog/s and/or cat/s, HH = household, No. = number

Not to allow dogs and cats to mate (41.6%) was the most common means of population control and the next are to give to someone or throw away newborns (8.0%), not to rear female dogs (7.3%), sterilizing (5.6%) and using local medicine (2.8). However, there are substantial numbers of people who do not know or practice any of the control methods (30.3%) or they do not need to control the dog and cat populations (4.3%). The majority of the interviewed households gave the newborn puppies and kitten to someone (77.9%), while the rest either kill/throw away (16.2%), not known because they do not own females (3.4%) or keep it/sale (2.5%). Households were also asked what they do suggest to control stray dogs and cats and 46.1% responded to educate the society not to release dogs and cats for stray and 42.9% to kill the stray, while 5.6% responded to collect back home and 1.3% to castrate and about 4.1% do not know the best option to suggest (Table 3).

**Table 3:** Population control of dogs and cats

Item	Category	No. of HH	Percent
Means of dog and cat population control	Not allow to mate	176	41.6
	Not known	128	30.3
	Give to someone/throw away	34	8.0
	Not to rear female	31	7.3
	Sterilize/ give drug	24	5.7
	No need to control	18	4.3
	Local medicine to sterilize	12	2.8
Action on new-born pet	Give to somebody	345	77.9
	Throw away or kill	72	16.2
	Not known/no female	15	3.4
	Keep it/sale	11	2.5
What do you suggest to control stray dogs and cats?	Educate society	281	46.1
	Kill stray dogs and cats	262	42.9
	Collect and manage them	34	5.6
	Not known	25	4.1
	Castrate not to mate	8	1.3

As shown in Table 4, among 213 households who do not keep dogs, 28.6, 26.3, 17.4, 12.7, 8.0, 4.2, and 2.8% gave dislike, fear of zoonosis, no time to devote, benefit not realized, financial problem, lack of private housing and lack of dog, respectively as the reason for not owning dogs. Similarly, among 371 households who do not keep cats, 25.3, 19.9 17.0%. 15.6, 10.0, 5.4, 3.8, and 1.6% gave shortage of cat supply, dislike, financial problem, no time to devote, benefit not realized, fear of zoonosis, lack of private houses, and allergy in the family, respectively and 1.3% of them do not know reasons for not owning cats. Three hundred ninety households who abandoned either dog or cat in their life claimed shortage of finance/feed (48.2%) as their major reason for abandoning, whereas the rest were bad behavior of dog and cat (21.5%), fear of zoonosis (5.1%), lack of time (1.5%), bite and legal issues (1.3%), and changing living area (0.8%), while 21.5% of the households do not know the reason for abandoning pets.

**Table 4:** Reason for not owning or abandoning pet in the three study towns

Items	Category	No. of HH	Percent
Reasons for not owning a dog	Hate/dislike	61	28.6
	Fear of zoonosis	56	26.3
	No time to devote	37	17.4
	Benefit not realized	27	12.7
	Financial problem / feed shortage	17	8.0
	No private houses	9	4.2
	Lack of dog	6	2.8
Reasons for not owning a cat	Shortage of cat supply	94	25.3
	Hate/dislike	74	19.9
	Financial problem / feed shortage	63	17.0
	No time to devote	58	15.6
	Benefit not realized	37	`10.0
	Fear of zoonosis	20	5.4
	Lack of private house	14	3.8
	Allergy in the family	6	1.6
	Not known	5	1.3
Reason for abandoning either dog or cat ^a^	Shortage of finance/feed	188	48.2
	Bad behavior of dog and cat	84	21.5
	Fear of zoonosis	20	5.1
	Lack of time	6	1.5
	The bite and legal issue	5	1.3
	Changing living area	3	0.8
	Not known	84	21.5

^a^Those owners who have either abandoned dog or cat

### Determinants of dog and cat ownership

The result of the logistic regression analysis for determinants of dog ownership was presented in Table 5. The multivariable logistic regression analysis showed a significant association of dog ownership with the town, community type, owning of other domestic animals, gender, age, and occupation of the head of the household (*p* < 0.05). Accordingly, the odds of dog ownership were greater in households of Ambo (OR = 2.1, 95% CI: 1.3, 3.4) and Bako (OR = 2.6, 95% CI: 1.5, 4.5) compared to Gojo town. The odds of dog ownership were greater in urban (OR = 1.8, 95% CI: 1.1, 2.8) than the peri-urban communities. Owning of other domestic animals had greater odds of dog ownership (OR = 2.5, 95% CI: 1.8, 3.7) compared to non-owner households. Households led by males had greater odds of dog ownership (OR = 2.4, 95% CI: 1.5, 3.8) compared to those led by females. The odds of dog ownership were greater in householders of age between 18 and 35 years (OR = 1.6, 95% CI: 1.0, 2.4) and in those older than 55 years (OR = 1.6, 95% CI: 1.0, 2.6) compared to 35–55 years of age groups. Among the different occupation groups, daily laborers were with higher odds of dog ownership (OR = 2.7, 95% CI: 1.1, 6.7) as compared to farmers. The other studied variables such as ethnic group, religion, level of education, family size, and marital status of the head of the household didn’t show significant association (*p* > 0.05).

**Table 5:** Logistic regression analysis of determinants for dog ownership in the three study towns

Variables	Category	No. Positive %	Univariable		Multivariable	
			OR (CI)	*p*-value	OR (CI)	*p*-value
Town	Gojo	61 (49.6)	1.0		1.0	
	Ambo	198 (64.9)	1.9 (1.2, 2.9)	0.004	2.1 (1.3, 3.4)	0.004
	Bako	138 (75.8)	3.2 (1.9, 5.2)	≤0.001	2.6 (1.5, 4.5)	0.001
Ethnic group	Oromo	295 (67.6)	1.0			
	Amhara	24 (72.7)	1.3 (0.6, 2.8)	0.548	–	–
	Gurage	10 (76.9)	1.6 (0.4, 5.9)	0.484	–	–
Community type	Peri urban	67 (49.3)	1.0		1.0	
	Urban	330 (69.6)	2.4 (1.6, 3.5)	≤0.001	1.8 (1.1, 2.8)	0.012
Owning other animals	No	160 (55.6)	1.0		1.0	
	Yes	237 (73.6)	2.2 (1.6, 3.1)	≤0.001	2.5 (1.7, 3.8)	≤0.001
Age of the household in years	36–55	146 (59.3)	1.0		1.0	
	18–35	153 (68.6)	1.5 (1.0, 2.2)	0.038	1.6 (1.0, 2.4)	0.033
	> 55	98 (69.5)	1.6 (1.0, 2.4)	0.047	1.6 (1.0, 2.6)	0.048
Gender of the household	Female	59 (49.6)	1.0		1.0	
	Male	338 (68.8)	2.2 (1.5, 3.4)	≤0.001	2.3 (1.5, 3.6)	≤0.001
Family size	< 4	144 (61.5)	1.0		1.0	
	4–6	108 (64.7)	1.2 (0.8, 1.9)	0.334	–	–
	> 6	145 (69.4)	0.9 (0.6, 1.3)	0.522	–	–
Level of education	Illiterate	55 (60.4)	1.0		1.0	
	Primary	100 (62.1)	1.1 (0.6, 1.8)	0.793	0.8 (0.4, 1.5)	0.463
	Secondary	120 (67.4)	1.3 (0.8, 2.3)	0.063	1.0 (0.5, 1.9)	0.968
	University	122 (67.8)	1.4 (0.8, 2.3)	0.231	1.3 (0.7, 2.3)	0.429
Occupation	Farmer	80 (60.2)	1.0		1.0	
	Self-emp.	168 (62.0)	1.1 (0.7, 1.6)	0.721	1.0 (0.6, 1.6)	0.894
	Gov. emp.	119 (71.3)	1.6 (1.0, 2.7)	0.044	1.4 (0.9, 2.4)	0.154
	Daily lab.	30 (76.9)	2.2 (1.0,5.0)	0.059	2.7 (1.1, 6.6)	0.025
Religion	Protestant	161 (61.7)	1.0			
	Muslim	7 (63.6)	1.1 (0.3, 3.8)	0.896	–	–
	Orthodox	224 (67.5)	1.3 (0.9, 3.8)	0.143	–	–
	Wakefata	5 (83.3)	3.1 (0.4, 27.0)	0.304	–	–
Marital status	Marital	347 (64.7)	1.0			
	Single	50 (67.6)	1.1 (0.7,1.9)	0.632	–	–

Wakefata = Oromo practice of believing in a creator being*,* Gov. = government, emp. = employee, lab = laborer

The same variables were computed for cat ownership as described in Table 6. However, the multivariable logistic regression analysis showed that study town, owning of other domestic animals, possessing dog/s, family size, and genders of the head of the households were significantly associated with cat ownership (*p* < 0.05). The odds of cat ownership were greater in households of Bako town (OR = 2.0, 95% CI: 1.2, 3.4) when compared to households in Gojo town. The odds of cat ownership was greater in households owning other animals (OR = 2.0, 95% CI: 1.4, 2.9) than non-owners. Owning dogs had greater odds of cat ownership (OR = 2.4, 95% CI: 1.6, 3.6) when compared to non-dog owners. As opposed to dog ownership, households led by females had greater odds of cat ownership (OR = 1.7, 95% CI: 1.1, 2.7) compared to those led by males. Families with members greater than 6 had greater odds of cat ownership (OR = 1.6, CI: 1.1, 2.5) as compared to families with 4–6 members.

**Table 6:** Logistic regression analysis of determinants for cat ownership in the three study towns

Variables	Category	No. Positive %	Univariable		Multivariable	
			OR (CI)	*p*-value	OR (CI)	*p*-value
Town	Gojo	37 (30.1)	1.0	–	1.0	–
	Ambo	112 (36.7)	1.3 (0.9, 2.1)	0.193	1.4 (0.9, 2.3)	0.165
	Bako	45 (49.4)	2.3 (1.4, 3.7)	0.001	2.0 (1.2, 3.4)	0.008
Ethnic group	Oromo	131 (35.4)	1.0			
	Amhara	13 (41.9)	1.2 (0.6, 2.5)	0.602	–	–
	Gurage	5 (71.4)	5.5 (1.5, 20.2)	0.011	–	–
	NR	45 (40.5)	1.0 (0.7, 1.6)	0.803		
Community type	Peri-urban	36 (27.5)	1.0		1.0	
	Urban	158 (40.7)	1.7 (1.1, 2.6)	0.009	1.3 (0.8, 2.0)	0.303
Owning other animals	No	70 (27.9)	1.0		1.0	
	Yes	124 (46.3)	2.2 (1.6, 3.1)	≤0.001	2.0 (1.4, 2.9)	≤0.001
Possessing dog	No	45 (23.6)	1.0		1.0	
	Yes	149 (45.4)	2.8 (1.9, 4.0)	≤0.001	2.4 (1.6, 3.6)	≤0.001
Age of the household in years	> 55	42 (35.3)	1.0	–		
	18–35	83 (38.8)	1.1 (0.7, 1.7)	0.721	–	–
	36–55	69 (37.1)	1.1 (0.7, 1.7)	0.663	–	–
Gender of the household	Male	150 (36.1)	1.0		1.0	
	Female	44 (42.3)	1.3 (0.8, 1.9)	0.261	1.7 (1.1, 2.7)	0.014
Family size	4–6	48 (32.4)	1.0		1.0	
	< 4	67 (33.8)	1.1 (0.7, 1.6)	0.782	1.0 (0.7, 1.6)	0.829
	> 6	79 (45.7)	1.7 (1.1, 2.6)	0.010	1.6 (1.0, 2.5)	0.029
Level of education	Illiterate	33 (36.3)	1.0			
	Secondary	67 37.6)	1.1 (0.6, 1.8)	0.825		
	University	70 (38.9)	1.1 (0.7, 1.9)	0.674		
	Primary	69 (42.9)	1.3 (0.8, 2.2)	0.306		
Occupation	Self-emp.	80 (34.2)	1.0			
	Farmer	43 (37.1)	1.0 (0.7, 1.6)	0.874	–	–
	Daily lab.	13 (39.4)	1.4 (0.9, 2.3)	0.123	–	–
	Gov. emp.	58 (42.6)	1.2 (0.6, 2.5)	0.575	–	–
Religion	Protestant	84 (37.7)	1.0			
	Orthodox	104 (37.3)	1.0 (0.7, 1.4)	0.926	–	–
	Muslim	6 (54.5)	1.9 (0.6, 6.3)	0.311	–	–
	Wakefata	0	Omitted	–	–	–
Marital status	Maried	169 (37.0)	1.0			
	Single	25 (40.3)	1.1 (0.7, 1.9)	0.610	–	–
Is there a child < 16 years?	No	28 (30.4)	1.0		1.0	–
	Yes	166 (38.8)	1.7 (1.1, 2.7)	0.028	1.5 (0.9, 2.4)	0.099

*NR* none responding, *Gov.* Government, *emp*. employee, *lab*. laborer

## Discussion

Knowledge of dog and cat populations is important for planning effective control of dog and cat borne zoonosis and population control. In this study, 65.1% of surveyed households owned dogs, and 39.2% of them own cats. This finding is a bit different compared to the previous reporting of 33% of urban and 75.5% of the pastoralist households own dogs from eastern Ethiopia [16]. Reports from other African countries show 82% dog and 4.1% cat ownership in Harare, Zimbabwe [19], 63% dog ownership in Kenya [20], and 88.9% dog ownership in Madagascar [21]. Reports from non-African countries including Japan (24.2%, [22]), Italy (33.0%) dog, and (13.0%) cat [23] and the United States (36.1%, [24]) showed lower ownership compared to reports from developing countries. The variation among the reports could be due to the difference in socio-cultural, economic, and attitude towards pet ownership. In Ethiopia, the majority of the households keep the dog for guarding purposes like other developing countries, and owners are less responsive to their dogs and cats probably due to lack of animal welfare legislation in Ethiopia.

In the present study, the mean number of dogs per dog-owning households (1.1) was lower. Similarly, a higher proportion of dog-owning households keep one dog (74.8%). Different estimates have been published from both African and non-African countries: Tanzania 2.2 and 40% [25], United States 1.7 and 63%, and Taiwan 1.6 and 69.5%. This variation could reflect the socio-cultural and geographic differences in the distribution of factors influencing at the household level in different corners of the globe.

In line with the present finding Downes et al. [26] from Ireland reported a clear preference of households for dog ownership (35.6%) over the cat (10.4%). According to these authors, the higher preferences of dogs over cats was explained by the fact that a dog has a greater dependence on, and interaction with, human households than cats, and therefore integrate more readily into the family social network. However, Freiwald et al. [27] from metropolitan Chicago reported findings different from the current study.

The human to pet ratio is often used as an indicator of canine or feline over-population. In the present study, the human to dog ratio was 6:1 and the human to cat ratio was 10:1. The highest human to the dog (5:1) and human to the cat ratios (8:1) as well as the higher proportion of dog and cat-owning households in Bako town may be attributed to more livestock and crop production, which uses dogs and cats for guarding and rodent control, respectively. In urban areas of Zambia, De Balogh et al. [28] reported that households kept dogs with a human to dog ratio of 45:1. In the semi-rural areas, households keep dogs with the dog to the human ratio of 7:1. Rinzin et al. [29] from Bhutan estimated humans to owned dogs ratio of 10: 1, whereas 5:1–6:1 was reported from urban places of Chile [30]. The variation in the dog and cat population in the different reports could be related to socioeconomic status and cultural differences among the countries.

Generally, there is little attention given to feeding, housing, and health care of dogs and cats in Ethiopia; hence the overpopulation of dogs and cats might be associated with high carrier rates of diseases, inadequate veterinary service, poor public awareness, close contact between dog, cat, and people, as well as poor housing, management, and hygienic practices. In the present study, 59.7% (*n* = 196) of the dogs and almost all the owned cats had outdoor access. This means that because no or little feed is provided, such dogs/cats will wander the whole day searching for their food and come back home during the evening. On the other hand, such dogs and cats are not secured and they have access to other free-roaming dogs and wild canids such as foxes and hyena during the night. The human to dog ratio reported in this study is for owned dogs; thus, the total dog to human ratio would be higher when un-owned/stray dogs are also considered. Pulczer et al. [31] explained the consequence of dog overpopulation posed to the community like physical risks to people, the transmission of infections to people, and scared members of their household, suggesting that the situation in Ethiopia might be even worse.

In the present study, the male to female sex ratio for the dog was 3:1 and nerly 1:1 (0.7:1) for cats, showing the presence of more male dogs than female and female cats than male. Male dog dominance was also reported in other countries such as 56–84% male dogs in Chile [30], 1.6:1 in Madagascar [21], and 2:1 in Thailand [32]. During the questionnaire, survey participants stated that they prefer males to females for the reason that female dogs have disturbing behavior during breeding periods by groups of male dogs. Besides, people prefer male dogs to avoid unwanted litters as well. Pal [33] described the reason for the dominance of male dogs is due to the high preference for male dogs, the higher mortality rates of female dogs, and the selective removal of females from the population during breeding periods to avoid unwanted pregnancies. Kitala et al. [20] explained that households in Kenya believed that male dogs make better guard dogs and hunters, thus the tendency to provide better husbandry practices for male dogs. In contrary to dogs, the study participants prefer female cats to males. According to the study participants, male cats, once they left home do not come back especially during the mating time, thus they prefer to keep female cats. Besides, the study participants mentioned the presence of higher demand than supply for male dogs and female cats, particularly in Bako town.

The estimated life expectancy of owned dogs was 12.3 years and that of cats was 9.2 years in this study. The mean life expectancy of mountain dogs in Switzerland was reported as 8.3 years [34]. According to these authors, generally, the average life expectancy of an animal is determined by genetic makeup, metabolic rate, body size, disease condition, etc. For instance, mountain dogs have a low life expectancy and the life expectancy of free-ranging dogs and outdoor cats is usually short because they are more likely to catch a disease or to suffer from some kind of trauma. In the developed countries, people may have a birth certificate for their dogs and cats, while in our case people simply estimate the age and they do not know the exact age of their pet. Thus, considering the disease burden and inadequate health care system, inadequate feeding, and housing of dogs and cats in Ethiopia, the relatively high estimated mean life expectancy of owned dogs and cats in the present study is not to our expectation.

This study showed that the dominant means of acquiring dogs and cats were from neighbors and family free of cost, which is in line with the findings in other countries [29, 30]. Reports from Chicago indicate that the main sources of acquisition of dogs were from a breeder or a shelter while that of cats was from a shelter or strays, and only a few were obtained as a gift from friends/family/neighbors [27]. Slater et al. [23] from Italy reported that the common source of cats was stray, gifts, and being born in the household. According to the present study, households used the sex, color, age, and breed of the dogs and cats for selection. Freiwald et al. [27] explained that pet owners want to select them with certain breed characteristics, both physical and behavioral, which is also applicable to the Ethiopian situation. The dominant purpose of dog ownership in the present study was for guarding the household; however, some reported the use of dogs for companionship or love and affection. This is similar to the findings of other researchers [29]. Likewise, most owned cats were used for the protection of property from mice and as companionship. This study revealed that dogs and cats in Ethiopia are primarily kept for guarding property and the house, unlike developed nations where they are primarily companion [35].

In Ethiopia, controlling free-roaming dogs and cat populations is an extremely challenging task. According to the present study, not allowing mating was the common means of controlling the pet population by the households, but a substantial number of households either do not know or do not need to practice any of the control methods. The newly born puppies and kittens obtained from uncontrolled breeding were either given to someone or thrown away. As an option to control stray dogs and cats, most study participants suggested educating the society, not to release their dogs and cats for stray and kill stray dogs and cats. However, in the developed world neutering of female dogs and cats was considered a major means of pet population control [26]. These authors also reported a 60% female dog and a 79% female cat castration rate in Italy, which would suggest a much lower dog and cat population growth in Italy. Nevertheless, it would be likely that the pet population growth will be high in countries like Ethiopia, where sterilization of dogs and cats is less commonly practiced.

The identified reasons for non-ownership of dogs in the present study include fear of zoonosis, hate and lack of time to devote and that of cats include the shortage of cat supply, hate, and shortage of finance/feed. Westgarth et al. [35] in the UK reported that the most common reasons for not owning a dog was due to working out all day, not enough time for the dog, and do not like dogs. The predominant reasons for abandoning dogs or cats in the current study were the shortage of finance to feed and their bad behavior. Several factors have been identified as determinants for abandoning dogs in other studies [36, 37]. Weng et al. [38] from Taiwan reported losing a dog due to behavioral problems of dogs such as barking and soiling public areas. The differences in the reasons for non-ownership among the studies might be due to the different study designs and the difference in the socioeconomic status of the studied communities.

Determinants of dog ownership assessed elsewhere [26, 28, 35] might not exactly fit in the Ethiopian context as ownership patterns might differ across countries due to cultural and religious differences. The significant explanatory variables for dog ownership in the final multivariable model were town where the households are residing, community type, owning of other domestic animals, age, and gender of the head of the household. A study from the UK identified factors such as ownership of a horse, age distribution groups, number of persons in the household, and the presence of adult females to be associated with dog ownership [35]. The economic situation of households appeared to play a major role in determining whether dogs were kept or not in urban areas [28]. Report from Ireland depicted human factors such as the presence of schoolchildren in the house and the presence of a pet cat in the house and gender and age for dog ownership and the presence of a dog in the house for cat ownership [39]. This is partially in line with the present study. In the present study, cat ownership was associated with the town where the households are residing, owning other domestic animals, possessing a dog, and the gender of the head of the household, which is also partially in accord with the aforementioned reports. When compared to women the issue of guarding the household property is more likely for men, leading to higher dog ownership in male-led households. In addition, the better attitude towards dog ownership by men than women might contribute to higher odds of dog ownership in male-led households [25]. Compared with men, women are more likely to feed and care for cats, leading to higher cat ownership in female-led households. This study of dog and cat ownership focused on three smaller towns of Ethiopia, so care is required when generalizing the results to other parts of Ethiopia or other countries because the socio-cultural situations might vary even within Ethiopia.

This study was the first to assess the demography and determinants of dogs’ and cats’ ownership in Ethiopia. The limitations of this study could be the failure to include a separate question to record individual dog and cat ages, selection bias that might have been introduced during door-to-door surveys, and difficulties in accessing some of the households. The low number of Muslims, few Amhara and Gurage ethnic groups, and a higher proportion of non-respondents made the comparison of religion and Ethnic groups less sound.

## Conclusion

This study provide insights into the determinants of ownership of dogs and cats and their characteristics in Ethiopia. Dog and cat ownership is common in the three studied towns of Ethiopia. Dogs are more commonly owned than cats. The dog and cat populations were relatively high in the study areas, the highest being in Bako town, with male dogs and female cats getting the more preferred sex groups owned. There was also a significant proportion of owned pets having access to an outdoor environment, implying risk to the owners as well as the society. It was also found that the means of obtaining, the reason for keeping and abandoning dogs and cats in the present study is partly different from those reported in the developed countries. Dog ownership was associated with study town, community type, owning of other domestic animals, gender, and age of head of the households. With minor differences, cat ownership was also associated with study town, owning other domestic animals, possessing dog, and gender of head of household. The results of this study could be used for the provision of veterinary services and for quantifying health risks and benefits associated with dog and cat ownership and control of pet population and zoonosis.

## Methods

### Description of the study towns

**Fig. 1:**
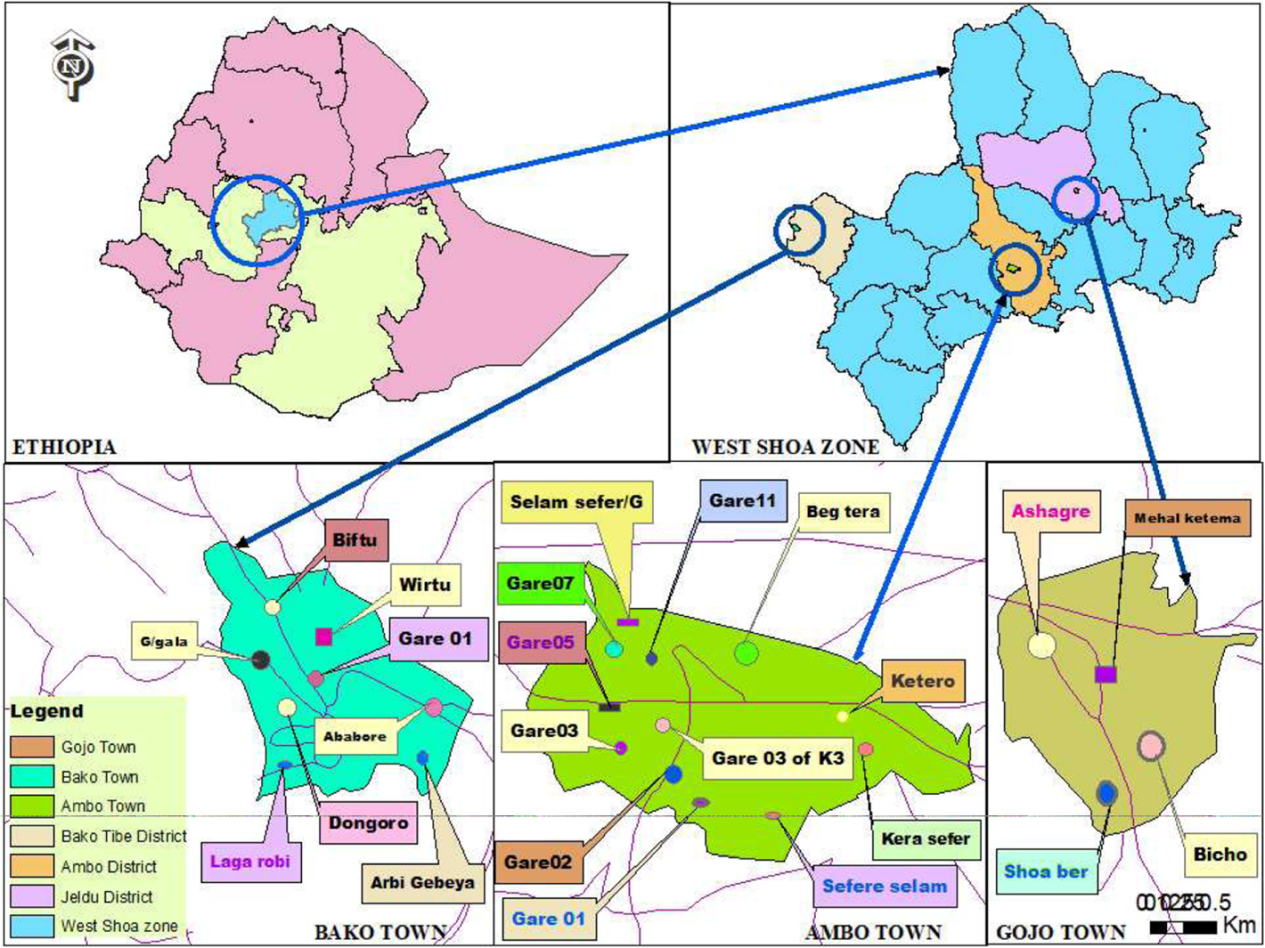
Map of the study towns. (Created by Arch map 10.2). The map shows the location of the three tows in Ethiopia and the different colored points in each town whose names are pointed from outside are those ‘*Gotes*’ (holding 20–30 households; subdivision of *kebeles*; the smallest formal administrative unit in the towns) that were selected and included in the study

The study was conducted in three selected district towns namely Ambo, Bako, and Gojo of West Shoa Zone, Oromia region, Ethiopia (Fig. 1). The three towns were selected to cover the different agro-ecological conditions in the zone. Ambo town is the administrative center of the West Shoa Zone located 114 km west of Addis Ababa. The town has a total human population of 63,733 and has midland altitude and moderate temperature. Bako town, the administrative center of Bako-Tibe district, is located 260 km West of Addis Ababa. The town has a total human population of 23,511 and has tropical temperatures and midland altitude. Gojo town, the administrative center of Jeldu district, is located 120 km West of Addis Ababa. The town has a total human population of 14,794 and has a highland altitude and colder ambient temperature [40]. There is no statistical information recorded on the dog and cat populations in the three towns.

### Study population

The study populations were the households residing in the three study towns both owning and not owning dogs and cats. Those households either owner of the pet (dog or cat) or not, who were a volunteer for the interview were included in the study. Since there is no dog and cat ownership registration in the country, ownership is verified during the questionnaire survey. A household was considered as the owner of a dog/cat if they claim ownership regardless of the provision of shelter, food, and health care. Those dogs and cats that are owned whether neglected or properly managed are considered in the study. However, those that do not have a home (stray) were not included in the study as this survey is household-based. Owned dog and cat populations of all age groups, sexes, breed, and management status found in the three study towns were recorded.

### Cross-sectional household survey

A cross-sectional household-based questionnaire survey was carried out from January 2015 to June 2017. To arrive at the required sample size to study households, Thrusfield, [41] formula was used. In the absence of an earlier study on the demography and determinants of dog and cat ownership in Ethiopia, 50% expected prevalence (P) and 95% confidence interval with a 5% desired absolute precision (d) was considered. The calculated sample size (*n* = 384) was raised to 610 to account for the non-response rate and design effect. The total sample size was distributed to the three study towns proportional to the human population: 305, 182, and 123 for Ambo Bako and Gojo respectively. A multi-stage sampling procedure was employed to select households in this study. Ambo, Bako, and Gojo towns have three, two, and one “*Kebeles*” (refers to the smallest administrative unit), respectively. All *Kebeles* of the study towns were included in the study. From each “*Kebeles*,” four “*Gotes*” (“*Gote*” is a subdivision of *Kebele* containing 20–30 households) were randomly selected using the list of *Gotes* in each *Kebeles* provided by local administrators. The index household in a *Gote* was randomly selected and subsequent households were surveyed door to door.

A structured questionnaire was developed based on the information gathered from the literature. The questionnaire originally prepared in English was later translated to “Afan Oromo” (regional working language). The questionnaire was administered by trained data collectors to randomly selected households and the questions were answered by heads or adult members (> 18 years) of the household. The questions included; the name of the district town, *Kebele, Gote*, community type (urban, peri-urban), socio-demographic data of respondent/head of household (age, sex, ethnicity, religion, marital status, occupation, and family size). Questions regarding dogs and cats demography included; the presence or absence, number, sex, breed, life expectancy, and living status (indoor/outdoor). Other characteristics such as means of acquiring, factors considered for acquiring, the method for population control, the fate of newborns, ownership of other domestic animals (cattle, sheep, goat, poultry, and other animals, the purpose of owning and reasons for not owning or abandoning dogs and cats were also included (Additional file 1).

### Data management and analysis

Data generated from the questionnaire survey were entered into Microsoft Excel spreadsheets, coded (Additional file 1), and analyzed using STATA version 14.0 for Windows (Stata Corp. College Station, TX, USA). Descriptive statistics (frequency, mean, ratio, and proportions) were used to summarize the data. Chi-square and one-way ANOVA were used to compare the variation in the the proportion and mean number of pets owned by households, respectively. Logistic regression analysis was used for the analysis of factors (independent variables) associated with dog and cat ownership status of the households (dependent variable). Univariable logistic regression was used to compute the crude odds ratio and *p*-values, then after those non-collinear variables with *p*-value < 0.25 and comparable frequencies were selected for multivariable logistic regression to identify predictors of dog and cat ownership. Odds ratio (OR) and the 95% confidence intervals (CI) were calculated and the level of significance of α = 0.05 was considered in all the analyses.

## Supplementary Information

**Additional file 1.** Questionnaire to investigate demography and determinants of dog and cats ownership. The questionnaire was developed based on the information gathered from the literature. The questions include the address and socio-demographic characteristics of the respondents, and the demography of dogs and cats. Moreover, means of acquiring and factors considered for acquiring, the method for population control, the fate of newborns, the purpose of owning, and reasons for not owning or abandoning dogs and cats were included.

## Data Availability

Datasets supporting the conclusions of this article will be available upon reasonable request from the corresponding authors.

## Abbreviations

CI: Confidence interval
OR: Odds ratio
Ave: Average
HH: Household
No: Number
