# Supplementary material for: Demography and determinants of dog and cat ownership in three towns of West Shoa zone, Oromia Region, Ethiopia
Source: BMC Vet Res. 2020 Dec 10;16:481. doi: 10.1186/s12917-020-02699-4 (PMC7730736; doi:10.1186/s12917-020-02699-4)
Supplement: Supplementary file 1 — Additional file 1. Questionnaire to investigate demography and determinants of dog and cats ownership. The questionnaire was developed based on the information gathered from the literature. The questions include the address and socio-demographic characteristics of the respondents, and the demography of dogs and cats. Moreover, means of acquiring and factors considered for acquiring, the method for population control, the fate of newborns, the purpose of owning, and reasons for not owning or abandoning dogs and cats were included. [file 12917_2020_2699_MOESM1_ESM.docx]

## Questionnaire survey to study demography and determinants of dog and cats ownership

**HH Code**-------------------- **Id No. of dogs**-------------------- **Id No. of cats**--------------------

1. **Respondents and ecological information**
2. Name of the respondent --------------------
3. District /town -------------------- Kebele-------------------- Sub-kebele/Goti/Village-------------------- Telephone-------------------- House no. --------------------
4. Ethnic group --------------------
5. Age of respondent --------------------
6. Sex of respondent--------------------
7. Community type: Urban 🗖 Peri-urban 🗖 Rural 🗖
8. Altitude-------------------- latitude -------------------- Min. average annual Temp. -------------------- Average annual Temp--------------------
9. Rain fall, (annual precipitation) --------------------
10. Season of the year --------------------
11. **Pet demographic and ownership patterns**
12. Do you have cat (s)? Yes 🗖 No 🗖
13. Do you have other domestic animals? Yes 🗖 No 🗖

If yes which one? Cattle 🗖 Sheep 🗖 Goats 🗖 Poultry🗖 Other animals’ --------------------

1. No. of cats, if present. Male -------------------- Female-------------------- Total --------------------
2. Do you have dog (s)? Yes 🗖 No 🗖
3. No of dogs, if present. Male-------------------- Female-------------------- Total--------------------
4. Breed of dog, if present: Indigenous breed 🗖 Exotic breed 🗖 Cross bred 🗖
5. Means of acquiring cat: Friends 🗖 Family 🗖 Neighbors 🗖 Street/ stray breeder 🗖Other- specify--------------------, --------------------, --------------------
6. Means of acquiring dog: Friends 🗖 Family 🗖 Neighbors 🗖 Street/ stray breeder 🗖 Other- specify--------------------, --------------------, --------------------
7. Which factors do you consider in selecting cat as pet animal? Age🗖 Sex🗖 Fur color 🗖 Breed 🗖 other (specify).
8. Which factors do you consider in selecting dog as pet animal? Age🗖 Sex🗖 Fur color 🗖 Breed 🗖 Other (specify).
9. Why do you keep dogs and cats? ( Purpose of keeping dog and cat)

Dog cat

- Companionship 🗖 🗖
- Love and affection 🗖 🗖
- For the benefit of the children 🗖 🗖
- Someone to greet me when I come home 🗖 🗖
- Protection of home or property 🗖 🗖
- Stimulus for me to exercise 🗖 🗖
- Hunting 🗖 🗖
- Guiding blinds 🗖 🗖
- Breeding value 🗖 🗖
- Other, specify-------------------- , --------------------, --------------------

1. Length of time of pet ownership (in years) --------------------
2. List benefits of pet ownership --------------------,--------------------, --------------------, --------------------, --------------------, --------------------
3. Estimated life expectancy of dog: --------------------
4. Estimated life expectancy of cats: --------------------
5. How do you control the breeding/ population of your dogs and cats? Not allow to mate🗖 To kill the young🗖 Other --------------------, --------------------, --------------------
6. What do you do with the newborns if your dogs and cats gives birth? Kill 🗖Give for somebody🗖 Throw away🗖 Other --------------------, --------------------, --------------------
7. What do you suggest to alleviate the problem of stray dogs and cats? To educate the society🗖 Kill stray dog🗖 Other --------------------, --------------------, --------------------
8. What are your reasons for not owning dog or cat?

Dog cat

- No time to devote 🗖 🗖
- Hate /dislike 🗖 🗖
- Financial problem 🗖 🗖
- Benefit not realized 🗖 🗖
- Fear of zoonosis 🗖 🗖
- Allergy in the family 🗖 🗖
- Other, specify--------------------,--------------------, --------------------

1. What do you think would be the reason that some people relinquish or abandon their dog or cats? --------------------, --------------------, --------------------

**Household related parameters**

1. Age of head of household (in years) --------------------
2. Sex of head of the household: Male 🗖 Female 🗖
3. Number of persons in the household --------------------
4. Level of education of owners:-

Dog Cat

Illiterate 🗖 🗖

Primary school 🗖 🗖

Secondary school 🗖 🗖

Tertiary /university 🗖 🗖

1. Are there children <16 years in the household? Yes 🗖 No 🗖
2. Gender of dog owner--------------------
3. Gender of cat owner --------------------
4. Occupation of head of the household: Government employee 🗖 Nongovernmental organization🗖 Self employee 🗖 Farmer 🗖

Other specify------------------------------------------------------------

1. Monthly income of household: --------------------
2. Is the respondent head of the household? Yes 🗖 No 🗖
3. Is there a member of the household who is allergic /asthmatic? Yes 🗖 No 🗖
4. Religion of the head of the household: Orthodox 🗖 Muslim🗖 Protestant 🗖

Other--------------------, --------------------, --------------------

1. Marital status: Married 🗖 Unmarried🗖
2. Way of life of cats: Totally indoor 🗖 Totally Outdoor 🗖 Mixed 🗖
3. Ways of life of dogs: Totally indoor 🗖 Totally outdoor 🗖 Mixed 🗖
